# A Fast and Effective System for Analysis of Optokinetic Waveforms with a Low-Cost Eye Tracking Device

**DOI:** 10.3390/healthcare9010010

**Published:** 2020-12-23

**Authors:** Chong-Bin Tsai, Wei-Yu Hung, Wei-Yen Hsu

**Affiliations:** 1 Department of Ophthalmology, Ditmanson Medical Foundation Chiayi Christian Hospital, Chiayi 62102, Taiwan; 00687@cych.org.tw; 2 Department of Optometry, College of Medical and Health Science, Asia University, Taichung 41354, Taiwan; 3 Department of Information Management, National Chung Cheng University, Chiayi 62102, Taiwan; 608530030@alum.ccu.edu.tw; 4 Center for Innovative Research on Aging Society, National Chung Cheng University, Chiayi 62102, Taiwan; 5 Advanced Institute of Manufacturing with High-tech Innovations, National Chung Cheng University, Chiayi 62102, Taiwan

**Keywords:** eye tracking, waveform analysis, optokinetic nystagmus, low-cost device

## Abstract

Optokinetic nystagmus (OKN) is an involuntary eye movement induced by motion of a large proportion of the visual field. It consists of a “slow phase (SP)” with eye movements in the same direction as the movement of the pattern and a “fast phase (FP)” with saccadic eye movements in the opposite direction. Study of OKN can reveal valuable information in ophthalmology, neurology and psychology. However, the current commercially available high-resolution and research-grade eye tracker is usually expensive. Methods & Results: We developed a novel fast and effective system combined with a low-cost eye tracking device to accurately quantitatively measure OKN eye movement. Conclusions: The experimental results indicate that the proposed method achieves fast and promising results in comparisons with several traditional approaches.

## 1. Introduction

Research on eye movement has provided a valuable source of information to vision science, psychology, and neurobiology [1,2,3]. There are numerous methods to measure eye movement, including electro-oculography, scleral search coil system, and video-oculography. In recent years, video-oculography has been used increasingly because of its advantages of non-invasiveness [4,5,6,7]. With the vigorous development of machine learning, more and more applications are also appearing in eye science [8]. The current commercially available high-resolution, research-grade eye trackers are often very expensive. The high cost and closed-source software often limit its wide clinical application. As low-cost video-based eye trackers are now increasingly available, there are emerging potentials in using these low-cost eye trackers in eye movement analysis and expanding the clinical application of eye movement analysis [7,9].

Optokinetic nystagmus (OKN) [10] is an involuntary eye movement induced by motion of a large proportion of the visual field. It consists of a “slow phase” with eye movements in the same direction as the movement of the pattern and a “fast phase” with saccadic eye movements in the opposite direction [11]. The launch of spoken term detection (STD) has a lot of signal processing content, including the range of dynamic time warping (DTW), and there are many directions for the detection of eye movement signals [12,13,14]. The neural pathways that mediate OKN extend from the retina foveal afferent pathway retina to the lateral geniculate body, occipital lobe, cerebellar flocculus, paramedian pontine reticular formation, and the efferent pathway of the ocular motor neurons. If there are damages along the neural pathways, the OKN response will be affected. OKN can be quantified by measuring the gain, which is the ratio of slow-phase eye velocity and stimulus velocity [15].

In this study, a novel fast and effective system is developed and combined with a low-cost eye tracking device to accurately quantitatively measure OKN eye movement. During eye movement recording, the human eyes often show unwanted noise or baseline drift due to fatigue or distraction, which interfere with the analysis of signals. Our algorithm can detect the position of FP and optimize the result by iterative signal modification. To validate the performance of the proposed system, the results of FP detection were comparable with those of manual reading by experienced physician and of several traditional approaches.

## 2. Materials and Methods

**Figure 1:**
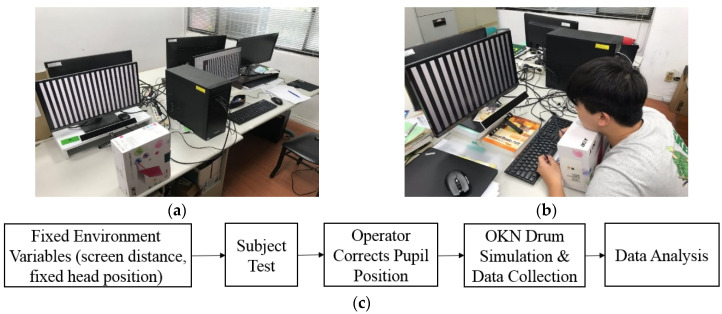
(**a**) Optokinetic nystagmus (OKN) data collection environment controlled by dual screens; (**b**) OKN detection is started after the head is fixed; (**c**) flowchart of experimental data collection.

In this study, we designed a dynamic stripe simulation program for OKN stimulation that is easy to adjust. We used the low-cost eye tracking device GP3 to collect OKN response data in a simple simulation environment and simply fixed the head to ensure the collected data would be free from interference. The GP3 eye tracker, which is produced by the Gazepoint company, is an easy-to-use, high-performance eye tracker. Gazepoint company produces the eye tracking system technology for everyone. The eye tracker comes with a software suite, Gaze point Control and Gaze point Analysis, which allows you to calibrate a participant, build an experiment, collect data, and analyze data. Finally, the collected data were used to get the position of the scanning moment by the FP filtering method. Figure 1 shows the operating environment.

### 2.1. Materials

Six healthy male adults were recruited in the study. The participants had no neurological, ophthalmological, or vestibular impairments. The six subjects underwent complicated tests, and the measured signals collected obvious OKN characteristics for these six subjects. Only participants with best-corrected visual acuity of 6/6 or higher on the Snellen scale were included in this study. The study was approved by the Institutional Review Board at the Ditmanson Medical Foundation Chiayi Christian Hospital and complied with the Declaration of Helsinki [16]. Participants gave informed written consent and were informed of their right to withdraw at any time.

Participants sat in a chair in front of the middle of a 23-inch LCD (Liquid Crystal Display) monitor (VZ2350HM, BenQ Corporation, Taipei, Taiwan). Distance between the head of the participant and the LCD monitor was set at 50 cm. The ophthalmologist recommended that the distance between the human eye and the computer screen should be 45–60 cm. Therefore, the setting of our environment uses a distance of 50 cm as a fixed value, and the width of the stripe animation is reversed according to this distance. To reduce the signal noise from vertical movement, the participants’ head was placed on a chin rest and a horizontal black bar of 0.5 cm height was placed at 3 cm below the vertical midline of the LCD monitor. The OKN stimulus was generated by showing moving black and white stripes on the LCD monitor at a frame rate of 60 Hz. Eye movements were recorded using a video-based eye tracker (GP3HD, Gazepoint, Vancouver, BC, Canada) that recorded eye movements with a maximum resolution of 30 arcmin at a sampling rate of 150 Hz, where arcmin is a unit of angular measurement equal to 1/60 of one degree.

The eye tracker was calibrated for each participant at the start of each procedure. Calibration was performed by fixating at five different positions on the monitor. The OKN was elicited with a square-wave grating alternative black and white vertical stripes of 4.6 cm width moving horizontally at fixed 20 cm/s speed. The participant was instructed to look at the moving patterns binocularly. Each test lasts for 30 s and was repeated three times. Measurements were recorded on a computer and stored for offline analysis. All programs and algorithms for analyzing data were developed in MATLAB (MathWorks, Natick, MA, USA).

### 2.2. Proposed OKN Eye Movement Measure System

After completing a series of tests and data collection, the dynamic SlopeThreshold and MoveThreshold were set up to screen out the eligible FP sections based on the data pre-processing and feature determination. The specific slope and length were used as the threshold, where SlopeThreshold represents the filtering range of the slope, which is used to control the OKN state of the eyeball according to the movement direction of the fringe, usually set positive or negative, and MoveThreshold stands for the distance the pupil moves, because the distance the pupil moves in the Fast Phase (FP) is larger, which is our index for screening. Finally, iterative corrections were made to fine-tune each eligible signal to the correct position.

**Figure 2:**
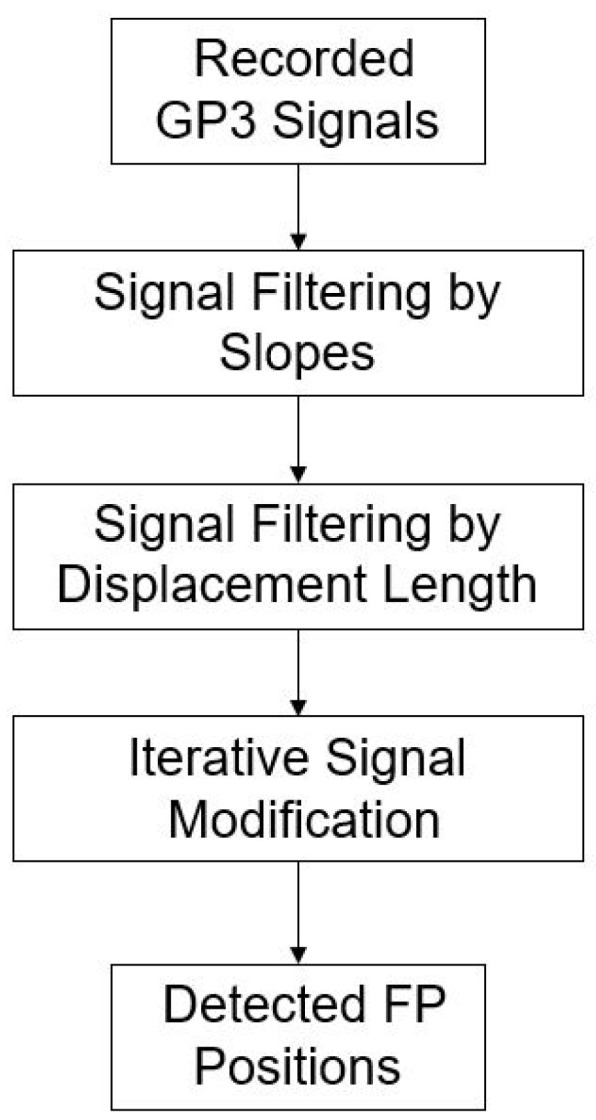
Flowchart of proposed fast phase (FP) position detection.

Using the slope determination method, we narrowed the selection range of FP in accordance with the occurrence conditions of FP formulated by Kanari et al. (2017) [17]. We found that only when the slope of the displacement between two points of FP was higher than a certain threshold could the occurrence condition set by Kanari be met. We selected the eligible points, compared their neighboring points to select the maximum/minimum, and repeated the iteration until it was located at the location where FP occurred. The process is shown in Figure 2. The pseudo code was as follows: the points in the matrix conform to the FP Slope; iteration count is the number of iterations compared with the adjacent points in the matrix; after iteration, the maximum/minimum is selected as the occurrence position of FP.

Since the GP3 tracking device can only take a maximum signal of 150 Hz and the data received are not stable and complete, even in the SP stage, involuntary eye movement can be incorrectly read as an FP, resulting in the need to dynamically determine the SlopeThreshold and dynamically set the MoveThreshold to filter FPs that are incorrectly read when nystagmus occurs in SP. The detailed processing steps (Algorithm 1) are as follows:
**Algorithm 1.** Proposed FP position detection.
1for i = 1: length (I)2 if (I’s slope meet S)3  Put Up/Down point to matrix;3 end4end5for j = 1: IC6 for i = length(M)7  gather points around peak (±1)9  find maximum/minimum of points10 end11endI:is the input dataS:is the slope thresholdM:is the matrix after select matching data from inputIC:is the iteration Counts (interationCount), through multiple iterations, the signal identified as the FP interval gradually moves to the highest and lowest points of FP

#### 2.2.1. Signal Filtering by Slopes

**Figure 3:**
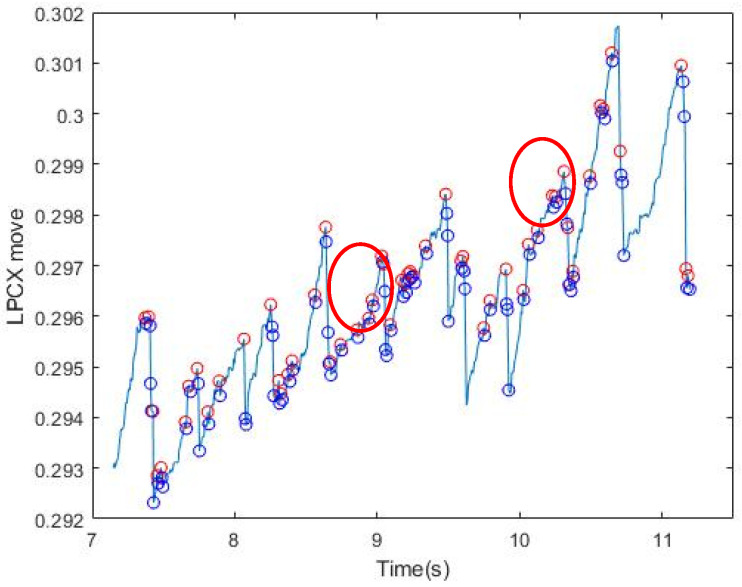
Results of signal filtering by slopes. All upper and lower values that meet the conditions of the relaxed FP slope are circled. The red circle shows the part of SP in which the slope also conforms to FP.

We narrowed the selection range of the signal according to the FP occurrence condition formulated by Kanari et al. (2017) [17]: The fast phase was detected using an eye velocity threshold of 20 deg/s. A slow phase velocity was calculated by averaging velocities for 50 ms just before each fast phase in each trial. We first screened for the slope at which FP occurred; however, the Hz collected by the GP3 device was low (60/150 Hz), and the points at which OKN occurred were not necessarily complete. Moreover, the FP conditions are strict, and the sampling point could be affected by the subject’s varying condition (dullness, fatigue, distraction, etc.). Some correct points would be lost if the conditions were strictly followed. Therefore, we started with the slope and relaxed the screening conditions, so as to extract all conditions under which FP could occur (Figure 3). It can be seen from the figure that in the SP interval, FP under the relaxed slope condition was also selected because of the uncertain condition of the subject, as mentioned above, or the uncontrolled natural nystagmus. Table 1 shows the results of filtering by slopes for six subjects. The next step was to filter misjudged FPs in SP Signal.

For accuracy strategy, generally 2 to 5 saccades occur in an OKN test per second. It varies with different subjects. We performed OKN tests on the left and right three times, respectively (each time about 30 s) for six subjects. According to the definition of FP formulated by Kanari et al. (2017), the highest point and the lowest point of each FP are manually marked as the position where FP occurred correctly. The formula for accuracy is defined as:

(1) $$Accuracy=\;\frac{\mathrm{Number}\;\mathrm{of}\;\mathrm{correct}\;\mathrm{detected}\;\mathrm{position}}{\mathrm{Number}\;\mathrm{of}\;\mathrm{all}\;\mathrm{manually}\;\mathrm{marked}\;\mathrm{position}}$$

The detected method is used to detect the FP position and is then compared with the ground truth.

#### 2.2.2. Signal Filtering by Displacement Length

The above condition for signal filtering by slopes is relaxed, but it can be dynamically adjusted. In this study, the subjects received the OKN test with a stripe that shifted to the right. For the convenience of sampling, we only sampled at a negative slope.

**Figure 4:**
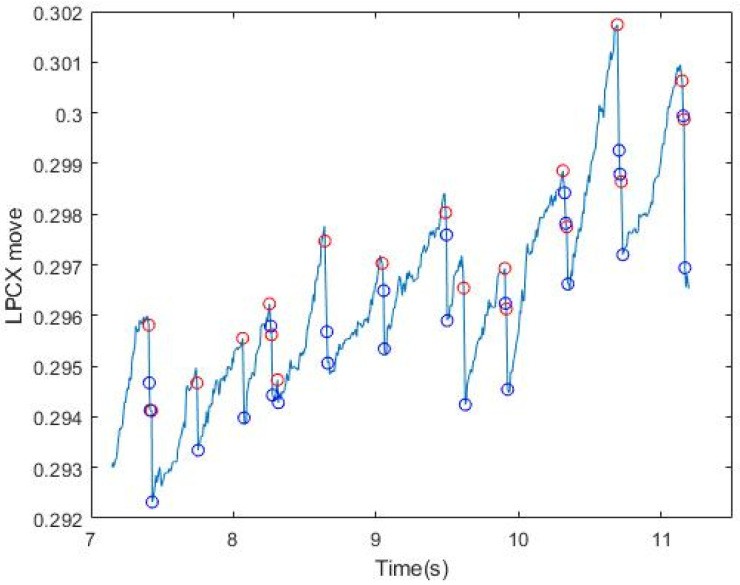
Results of signal filtering by displacement length. The red circle indicates the initial point of conforming to the slope and length, and the blue circle indicates the end point of conforming to the slope and length.

After collecting all the points at which FP occurred, we set a certain length (the maximum of FP between two points in SP) to filter uncontrolled nystagmus. Based on the displacement of length as the threshold, we used an FP length greater than Thr to filter the unsmooth signal caused by nystagmus (Thr (threshold) refers to the setting of MoveThreshold). After multiple tests by several subjects, it was found that the X-coordinate movement of the eyeball in GP3 exceeds Thr = 0.0006, which has a very high possibility for the FP interval. It can be adjusted slightly (Figure 4). It can be seen that after the length filtering, the points conforming to the FP condition were generally screened. Table 2 shows the results of filtering by slopes and displacement length for six subjects. The next step was to push them back to the highest and lowest points where FP occurred. In (3), the use of iterative modification is introduced.

#### 2.2.3. Iterative Signal Modification

**Figure 5:**
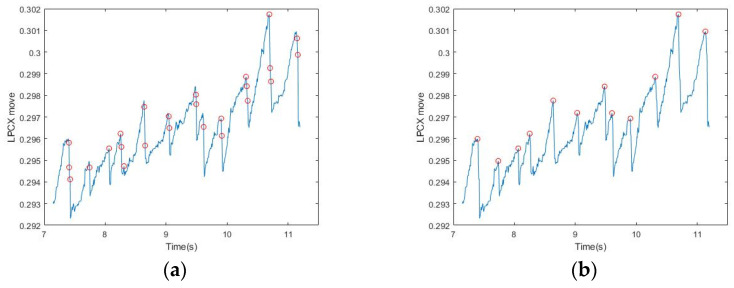
Results of iterative signal modification: (**a**) The starting point conforming to the length and slope in FP; (**b**) the maximum value obtained after comparing all starting points with their neighboring points and returning to the highest starting point.

As shown Figure 5a, although all the points chosen occurred at the interval positions of FP, they were not necessarily the highest and lowest points at the time of occurrence. The reason was that the initial changes of the eyeball may be instantaneous and small and may include nystagmus and other factors, therefore the peak may not be accurately obtained. Compared with the uncertainties that occur continuously in the SP interval, the occurrence of FP is instantaneous, therefore it is not affected by nystagmus and other factors. Thus, we could get the maximum/minimum value by comparing all the points with their adjacent points (adjacent ±1), and then make an iterative correction to the highest/lowest point (Figure 5). More iterations would result in greater accuracy. In this study, when there were more than eight observed iterations, all points returned roughly to the moment when FP occurred. Table 3 shows the final results of iterative signal modification after the previous steps for the six subjects.

## 3. Experimental Results and Discussion

### 3.1. Quantitative Measurement of Elicited OKN

**Figure 6:**
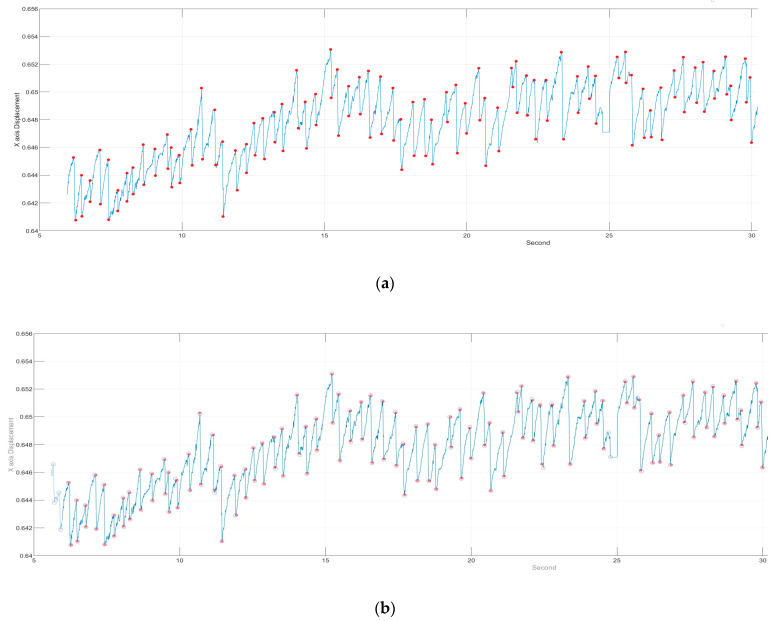
Results of the proposed method: (**a**) Results marked by the physician; (**b**) the comparison of the results of this method and those marked by the physician.

**Figure 7:**
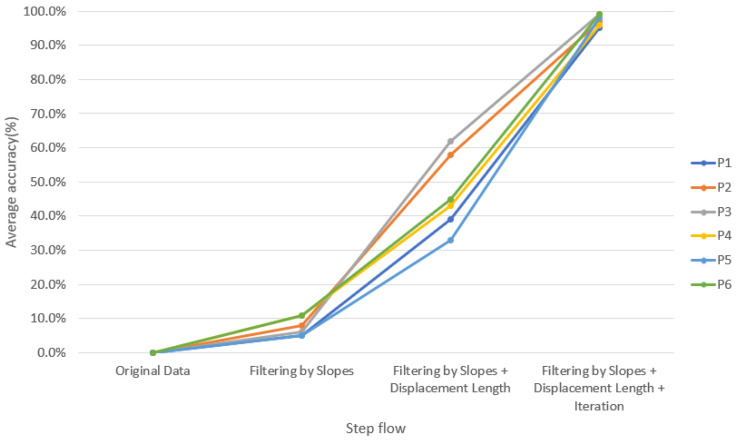
Average accuracy of each step for six subjects. (P1–P6 mean subjects 1–6).

In order to verify the accuracy of our method, we invited physicians to assist in marking the correct point when FP occurred and created a graph for comparison (Figure 6). For verification strategy, we randomly selected the OKN test data from each of the six subjects twice to allow the professional doctors to manually mark the location of the FP occurrence. Next, each intermediate process of our method was used to detection the FP location. For comparison, the position we circled contains the position marked by the doctor (red dot), which is considered to be the correct selection of the FP position, as shown in Figure 6b. Table 4 lists the average results of each step for six subjects and also lists *p*-values using paired t-test between each intermediate process of our method and ground truth. More specifically, the paired t-test was used to estimate whether the differences between the performance of each intermediate process of our method and ground truth were significant or not. The results showed that 97% of the FP occurrence points of OKN could be found through our method (Figure 7). The reason why they could not be completely identified was that some special signals were difficult to filter, which is explained in detail in Section C. For normal OKN signals, the location of occurrence could be completely obtained.

### 3.2. Comparisons with the State-of-the-Art Approaches

**Figure 8:**
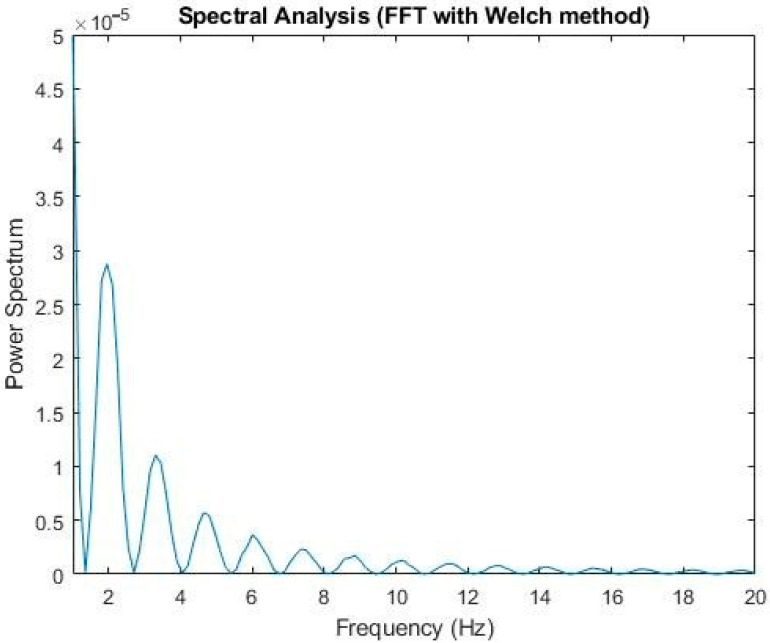
Based on the Fourier transform, Welch method, and Hamming window, the maximum influence spectrum was about 2 Hz to 4 Hz.

The traditional method adopted the Fourier transform combined with band-pass and high-pass filtering and employed the Welch method [18] to analyze the spectrum (2). It was found that the OKN frequency was about 2 Hz to 4 Hz (Figure 8). This frequency, compared with the GP3 callback, was consistent with the saccadic state of the subjects; that is, under our experimental environment, the subjects’ occurrence of OKN varied from two to four times per second

(2) $$P_{xm,M}\left(\omega_{k}\right)=\frac{1}{M}{\left|FFT_{N,k}\left(x_{m}\right)\right|}^{2}≜\frac{1}{M}{\left|\overset{N-1}{\underset{n=0}{\sum }}x_{m}\left(n\right)e^{-\frac{j2\pi nk}{N}}\right|}^{2}$$

**Figure 9:**
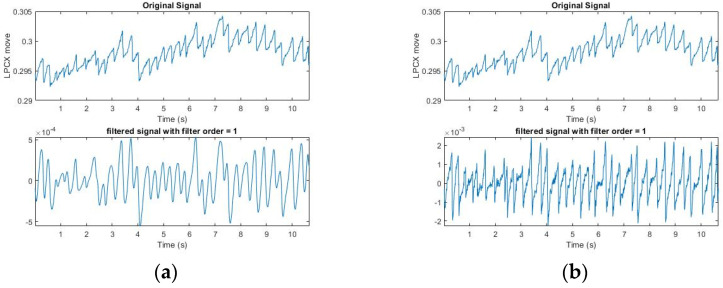
(**a**) The lower part represents the signal after the band pass; (**b**) the lower part represents the signal after the high pass. It was easier to mark the position of FP with the results of the high pass, but the set threshold was difficult to fully meet the conditions.

The Fourier transform was then used to find the frequency (3) at which FP occurred. High-pass and band-pass FIR filtering were adopted for peak detection. After smoothing, an adjustable threshold was set for the screen, and the filtered peak was found. The adjacent maximum/minimum values were found according to the signal position (Figure 9) [19]

(3) $$S\left(t\right)=\frac{a_{0}}{2}+\overset{N}{\underset{n=1}{\sum }}\left(a_{n}\cos\left(2\pi \frac{n}{T}t\right)+b_{n}\sin\left(2\pi \frac{n}{T}t\right)\right)\;\;$$

**Figure 10:**
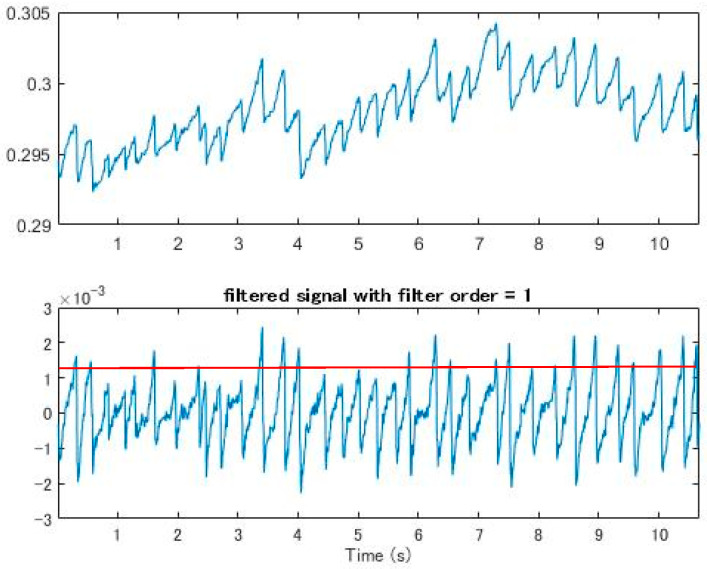
The frequency range selected after filtering. The upper part is the original signal, and the lower part is the signal after the band pass. The red line is the peaks that mark the height of the OKN, which needed to be determined manually and was not accurate.

This method ensured a certain degree of accuracy, but the parameters of the threshold needed to be adjusted every time. If the parameters were wrong, it would be possible to omit the FP position. Only after repeated tests by professionals could the setting become more accurate (Figure 10). Moreover, it could be easily affected by dullness and excessive eye movement, which make it difficult to set the threshold.

**Figure 11:**
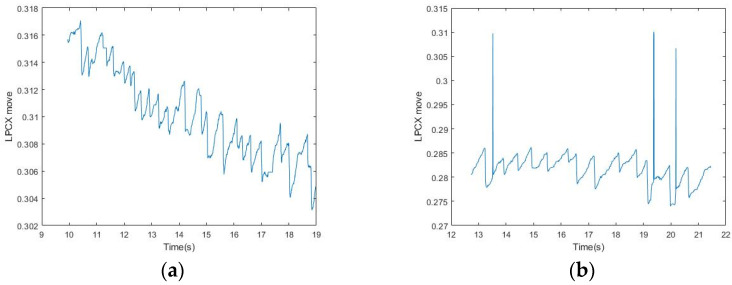
(**a**) Inability to focus on the same level and excessive drop from the starting position because of eye fatigue; (**b**) involuntary eye drift and signals of excessive displacement.

In the bandpass processing, although subjects were asked to focus on the fixed range of the screen, their eyes were difficult to control when they were tired, which caused eye drift, dullness, and other behaviors. Therefore, even if OKN occurred regularly, the eyes could not be fixed in the same range, and the subjects could easily be distracted (Figure 11). This phenomenon made it difficult to find the maximum amplitude of the signal either through high-pass or band-pass filtering. In addition, the response of FP in OKN was not consistent, and the amplitude of eye displacement was unstable. All these conditions made it difficult to find the highest (low) point of the fixed frequency in either high-pass or band-pass processing.

**Figure 12:**
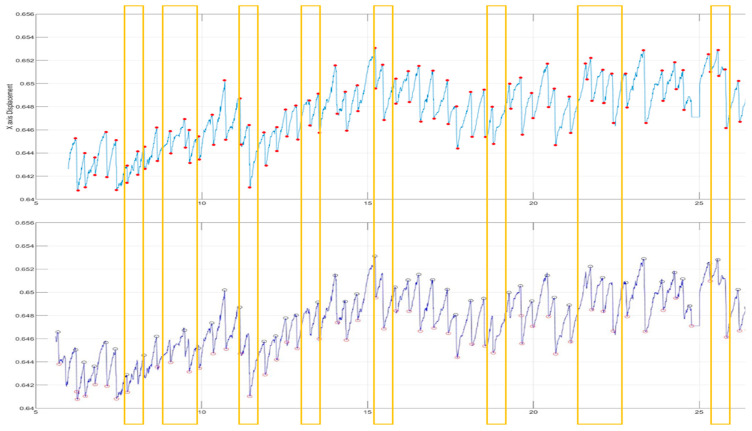
After the high-pass filtering and threshold setting, the yellow box represents the interval where FP points are not captured.

After the Fourier transform, we analyzed the high-pass and band-pass signals, and then set the threshold to compare with the signals marked by the physician after artificial observation. It was found that although the threshold was set very close to the maximum average height, there would still be omissions when the signal was elongated, and it varied greatly. As the eye moved away from the original position, it became more difficult to set the threshold and it was not necessarily accurate, which also led to the decrease of accuracy (Figure 12).

Under the complete dynamic signal, it could be found that an unstable signal would inevitably affect the FP judgment, and the screening based on the slope and amplitude of vibration had a higher identification rate than the band-pass (or high-pass) filtering after the Fourier transform. The reason was that the slope of FP would not affect the threshold judgment with the involuntary eye shift of the subject. Our method could eliminate the need for complex calculations and make the judging simpler and more accurate.

In order to evaluate the performance of the proposed method, the three traditional methods [6,8] were implemented for comparisons. Table 5 shows the comparison and analysis between the proposed method and the three traditional methods [6,8], and *p*-values using paired t-test between the three traditional methods and the proposed method. More specifically, the paired t-test was used to estimate whether the differences between the performance of three traditional methods and that of the proposed method were significant or not. At first, we thought that we should only use Peak finding [20] to find the instantaneous high point of FP. However, this method is prone to noise interference and can cause serious misjudgment, resulting in low accuracy. The application of the Fourier transform and filtering [19] depends on the judgment of values, which requires experienced researchers to select and set. Among them, high-pass filtering is the easiest to select, so the FP position can be correctly determined.

Regardless of the normal OKN identification rate, the uncontrolled noise produced by the subjects affected the determination of the correct FP during the prolonged test time. The OKN with noise includes dullness, fixation position deviation, and other phenomena, which further deepen the difficulty of filter setting and greatly decrease the accuracy. Our method simply filtered by the slope and moving distance, which solved many judgment problems, and screened the FP position from an intuitive angle. It reduced the influence of fixation deviation, leaving only the stagnant fixation problem (the original problem is explained in IV). In addition to the normal FP judgment of OKN and FFT combined with a high-pass filter that can be used well, there were good results on the correct FP judgment of OKN signals with noise.

### 3.3. Research Contributions and Limitations

Clinical detection of OKN often requires costly instruments, which poses considerable difficulties in clinical applications. Our main contributions were as follows: (1) low-cost instruments could be used to detect OKN, and (2) simple conditional screening and iteration could be used to obtain the accurate FP position, which was conducive to the calculation and analysis of the subject’s eye disease status.

**Figure 13:**
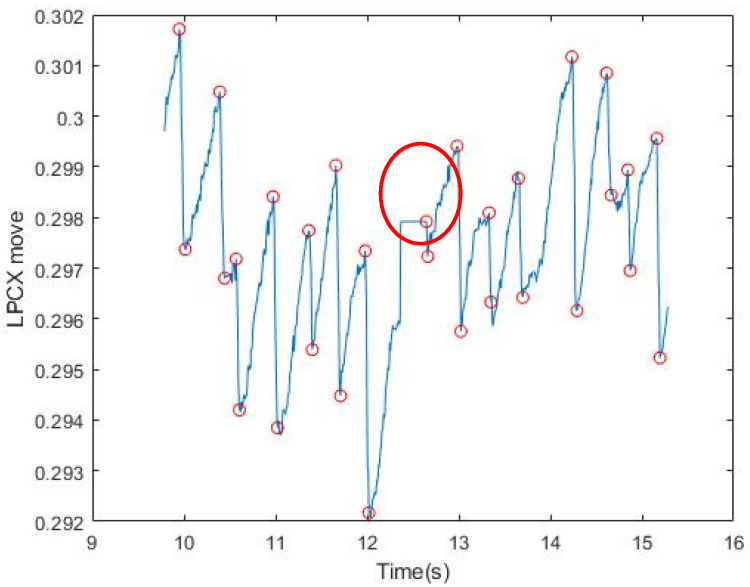
The red circle indicates a signal of the dull eye and that the FP determination has not been filtered.

Our method could be used to completely identify the FP position under stable conditions without large eyeball stagnation. However, the reaction of the eyeball cannot be controlled, and abnormal nystagmus and dullness are randomly generated and not included in FP/SP for reference (Figure 12). Our method has not yet been able to filter these unstable signals. If these phenomena occur more frequently, the accuracy of FP signal identification will decrease (Figure 13).

## 4. Conclusions and Future Work

In the field of neuro-ophthalmology, many scholars have tried to use OKR to study the physiological and pathological phenomena of eye movements (Aleci, Scaparrotti, Fulgori, & Canavese, 2017; Costa, 2011; Garbutt et al., 2004; Han et al., 2011; Hyon et al., 2010; Sung, Changhoon, Sunyoung, Jeong-Min, & Mo, 2017; Wester, Rizzo, Balkwill, & Wall, 2007). In the 1940s, scholars studied the relationship between OKR and VOR (Henderson, 1947; Henderson & Crosby, 1952). Sangi et al. (2015) tested and analyzed the data of children [3], finding that children are more susceptible to the influence of the external environment and that uncontrollable situations make it difficult to collect data, providing good reference value for the patients we may meet in future clinical applications. At present, FP judgment can be made for the source of stable signals, and the test model of Larrazabal et al. (2019) [21] has been used to simulate the OKN of healthy people [3].

The data collected by the six subjects in this experiment will be used as a reference index for future clinical eye disease patients. In future work, we will increase the screen distance, stripe animation changes, and other variables to test the patients next to the bed. With diversified tests, OKN factors that may affect patients with eye diseases will have the opportunity to be discovered. The high-accuracy FP recognition rate is of great help to the subsequent calculation of mean slow-phase velocity (MSPV) and other eye movement analysis parameters, and is beneficial to use as a reference index for patients with strabismus and other eye diseases.

## Figures and Tables

**Table 1:** Accuracy of filtering by slopes for six subjects.

Filtering by Slopes	
Subjects	Accuracy (%)
1	0.05
2	0.08
3	0.06
4	0.11
5	0.05
6	0.11

**Table 2:** Accuracy of filtering by slopes and displacement length for six subjects.

Filtering by Slopes and Displacement Length	
Subjects	Accuracy (%)
1	0.39
2	0.58
3	0.62
4	0.43
5	0.33
6	0.45

**Table 3:** Accuracy of iterative signal modification after the previous two steps (our method) for six subjects.

Iterative Signal Modification After the Previous Steps (Our Method)	
Subjects	Accuracy (%)
1	0.95
2	0.97
3	0.99
4	0.96
5	0.98
6	0.99

**Table 4:** Average Accuracy of Each Step for Six Subjects.

Average Accuracy (%)			
	Filtering by Slopes	Filtering by Slopes and Displacement Length	Our Method
mean	0.07	0.47	0.97
*p*-value	<0.001	<0.001	>0.01

**Table 5:** Identification rate of each method in identifying OKN signals and OKN signals with noise.

Methods	Normal OKN Signals	*p*-Value	OKN Signals with Noise	*p*-Value
Peak Finding [8]	0.43	<0.001	0.35	<0.001
FFT plus Band-Pass Filter [6]	0.96	<0.05	0.86	<0.01
FFT plus High-Pass Filter [6]	1	>0.05	0.91	<0.05
Proposed Method	1	-	0.97	-
